# Mesoporous silica nanoparticles for ^19^F magnetic resonance imaging, fluorescence imaging, and drug delivery†
†Electronic supplementary information (ESI) available: Detailed synthetic procedure, experimental procedure and Fig. S1–S7. See DOI: 10.1039/c4sc03549f



**DOI:** 10.1039/c4sc03549f

**Published:** 2014-12-23

**Authors:** Tatsuya Nakamura, Fuminori Sugihara, Hisashi Matsushita, Yoshichika Yoshioka, Shin Mizukami, Kazuya Kikuchi

**Affiliations:** a Division of Advanced Science and Biotechnology , Graduated School of Engineering , Osaka University , 2-1 Yamadaoka , Suita , Osaka 565-0871 , Japan . Email: kkikuchi@mls.eng.osaka-u.ac.jp ; Fax: +81-6-6879-7875 ; Tel: +81-6-6879-7924; b Immunology Frontier Research Center (IFRec) , Osaka University , 2-1 Yamadaoka , Suita , Osaka 565-0871 , Japan

## Abstract

Multifunctional mesoporous silica nanoparticles (MSNs) are good candidates for multimodal applications in drug delivery, bioimaging, and cell targeting. In particular, controlled release of drugs from MSN pores constitutes one of the superior features of MSNs. In this study, a novel drug delivery carrier based on MSNs, which encapsulated highly sensitive ^19^F magnetic resonance imaging (MRI) contrast agents inside MSNs, was developed. The nanoparticles were labeled with fluorescent dyes and functionalized with small molecule-based ligands for active targeting. This drug delivery system facilitated the monitoring of the biodistribution of the drug carrier by dual modal imaging (NIR/^19^F MRI). Furthermore, we demonstrated targeted drug delivery and cellular imaging by the conjugation of nanoparticles with folic acid. An anticancer drug (doxorubicin, DOX) was loaded in the pores of folate-functionalized MSNs for intracellular drug delivery. The release rates of DOX from the nanoparticles increased under acidic conditions, and were favorable for controlled drug release to cancer cells. Our results suggested that MSNs may serve as promising ^19^F MRI-traceable drug carriers for application in cancer therapy and bio-imaging.

## Abstract 2

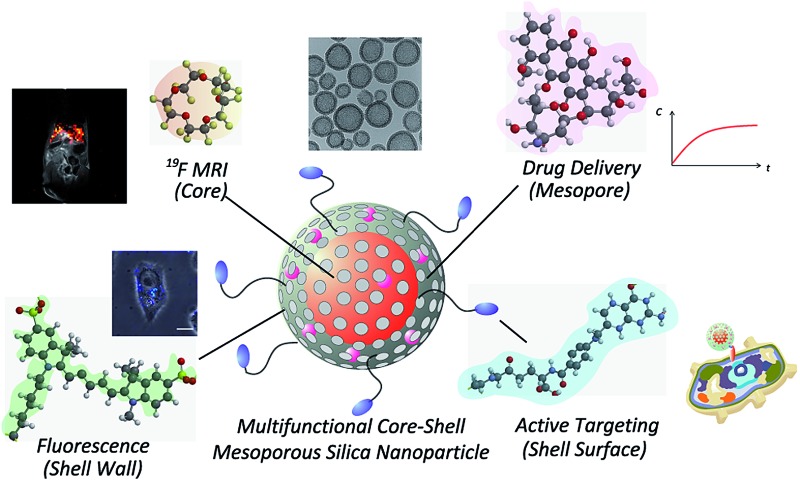
We described perfluorocarbon encapsulated in mesoporous silica nanoparticles which enabled dual modal imaging (NIR/^19^F MRI) and drug delivery.

## Introduction

Efficient delivery of drugs to diseased tissues is a major goal in the field of drug delivery in an effort to reduce adverse effects.^1^ Toward this end, various nanoparticle-based drug carriers such as liposomes, polymers, and inorganic materials have been developed.^2^ Among these nanocarriers, mesoporous silica nanoparticles (MSNs) have attracted significant attention owing to their attractive properties such as extremely large surface areas (1000 m^2^ g^–1^), tunable pore sizes (1.5–10 nm), and ease of functionalization *via* various synthetic approaches.^3^ Since controlled release of drugs from the pores of MSNs results in prolonged drug efficacy, MSNs serve as ideal materials for drug delivery.^4^

To assess the drug efficacy and toxicity of drug carriers, it is essential to monitor the localization of the drug carrier. Accordingly, MSNs modified with imaging agents have been developed. Fluorescence-traceable MSNs are useful for visualizing cellular localization *via* fluorescence microscopy. In particular, near infrared (NIR) fluorescent dye-modified MSNs are powerful nanomaterials for visualization in cells and *in vivo* localization.^5^ Magnetic resonance imaging (MRI)-traceable MSNs have also attracted attention in the field of drug delivery owing to their deep tissue imaging capabilities. MRI is a non-invasive *in vivo* molecular imaging technique used in both clinical- and research-based fields.^6^ Gd^3+^ complex- or superparamagnetic iron oxide (SPIO)-loaded MSNs are widely utilized owing to their high sensitivity.^7^ Recently, multimodal imaging techniques (NIR/MRI) have gained attention because the combination of NIR and MRI provides detailed information regarding deep tissues and cell localization.^8^ Therefore, MSNs that can be traced *via* multiple techniques (NIR/MRI) are desired.

^l9^F MRI has attracted significant attention owing to the high sensitivity comparable to that of ^1^H and negligible background signals.^9^ ^19^F MRI contrast agents are suitable for tracking specific biological makers.^10^ Although MSNs loaded with 19F-traceable fluorine compounds have been developed, the fluorine-containing compounds can diffuse from the pores of the MSNs.^11^ In contrast, perfluorocarbon (PFC)-encapsulated micelles have emerged as highly sensitive ^19^F MRI contrast agents and have been utilized as cell-tracking markers.^12^ Although PFC encapsulated micelles with potential for use in drug delivery have been reported,^13^ nanoparticles are not suitable for use as drug carriers owing to their low stability in aqueous solutions. Thus, MSNs with PFC-based cores are potentially viable for efficient drug delivery and as traceable drug carriers by ^19^F MRI.

In a previous study, we developed novel, highly sensitive ^19^F MRI contrast agents termed FLAME (FLuorine Accumulated silica nanoparticles for MRI Enhancement), composed of a PFC core and amorphous silica shell.^14^ FLAME has excellent properties such as high sensitivity, feasible surface modifications, and biocompatibility. Furthermore, we showed that FLAME was useful for cell labeling and *in vivo* tumor imaging.^14^ In this study, by advancing the silica coverage of the PFC core, we developed ^19^F MRI traceable MSNs as drug carriers. The MSNs consisted of the PFC core and an NIR dye modified-mesoporous silica shell, enabling both dual modal imaging (NIR/^19^F MRI) and drug delivery. The modification of targeting ligands on the MSN surface enhanced the internalization of the MSNs into tumor cells, resulting in adequate drug efficacy due to fast drug release in acidic environments.

## Results and discussion

### Design, preparation, and physical properties of ^19^F MRI- and fluorescence-traceable drug delivery carrier

**Fig. 1:**
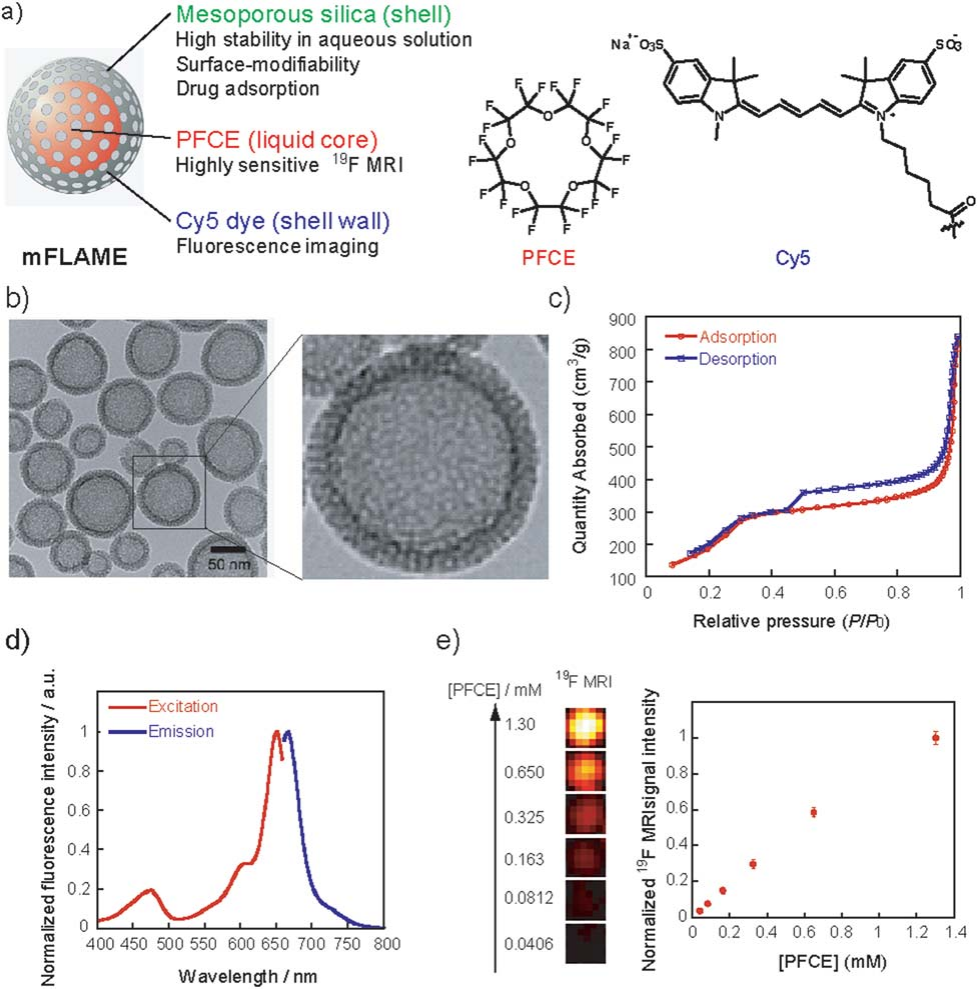
(a) The components of mFLAME. (b) Transmission electron microscopy (TEM) image of mFLAME. (c) N_2_ adsorption/desorption isotherms of mFLAME. (d) Excitation and fluorescence spectra of mFLAME in phosphate buffered saline (pH 7.4). (e) (Left) ^19^F MRI of mFLAME in phosphate buffered saline (500 μL); (right) plot of normalized ^19^F MRI signal intensity *versus* PFCE concentration.

For efficient drug delivery and monitoring of drug carriers, we designed a novel drug carrier with dual modal imaging capabilities (NIR/^19^F MRI), termed mFLAME (mesoporous FLAME, Fig. 1a). mFLAME consisted of a perfluoro-15-crown-5-ether (PFCE) core and mesoporous silica shell. PFCE is a highly sensitive ^19^F MRI marker owing to its twenty magnetically identical fluorine atoms.^12^ Mesoporous silica shells are stable in aqueous solutions, and drugs can be loaded into their pores. Furthermore, active targeting to foci can be achieved by modifying targeting ligands on the mFLAME surface. To impart fluorescence imaging capabilities, Cy5 dye was covalently modified on a mesoporous silica shell by silica polymerization in the presence of Cy5-conjugated 3-aminopropyltriethoxysilane (APTES).

The procedure used to prepare mFLAME is shown in Scheme S1.† Generally, PFCE requires the use of surfactants for biological applications owing to its extremely low water solubility.^12^ We discovered that *n*-cetyltrimethylammonium bromide (CTAB), which is commonly used for the synthesis of MSNs, was capable of dispersing PFCE in water. Furthermore, the core–shell type nanoparticles that constitute the PFCE core and that of the mesoporous silica shell could be produced from the PFCE emulsions by a sol–gel process.

Characterization of the nanomaterials was carried out using dynamic light scattering (DLS). The *ζ* potential and hydrodynamic diameter of the PFCE emulsions were +51.0 mV and 78 nm, respectively. In contrast, those of mFLAME were –21.1 mV and 165 nm, respectively. The DLS data revealed that mFLAME did not form aggregates in the aqueous solution. The increase in size and decrease in *ζ* potential as compared to those of the PFCE emulsions were due to the formation of the silica shell. Transmission electron microscopy (TEM) revealed that mFLAME had mesopore and core–shell structures, and the average diameter of the particles was 79 ± 20 nm (Fig. 1b). The N_2_ adsorption/desorption isotherms of mFLAME revealed a typical mesoporous structure with a Brunauer–Emmett–Teller (BET) surface area of 715 m^2^ g^–1^, pore volume of 1.21 cm^3^ g^–1^, and pore width of 6.7 nm (Fig. 1c). Fluorescence measurements confirmed that mFLAME could be traced by NIR fluorescence (Fig. 1d).

Next, ^19^F NMR was measured to confirm whether PFCE is encapsulated in mFLAME. The ^19^F NMR of mFLAME showed a single sharp peak at –16.4 parts per million (ppm), whose chemical shift was consistent with that of PFCE (Fig. S1†). The transverse relaxation, *T*_2_, of mFLAME was 0.211 s, which was almost the same as that of the PFCE emulsion (*T*_2_ = 0.242 s). As such, the silica coating did not affect the ^19^F MRI sensitivity of PFCE. ^19^F MRI measurements using capillary phantoms revealed strong ^19^F MRI signals from the phantoms of mFLAME, and the ^19^F MRI signal intensity increased according to the concentration of PFCE (Fig. 1e). *In vivo* MRI was performed following the injection of carboxylated mFLAME (mFLAME–COOH) (Scheme S2†) into a living mouse. The ^19^F MRI signals of mFLAME were detected in the liver, indicating that mFLAME had sufficient sensitivity for *in vivo* ^19^F MRI applications (Fig. S2†).

### Specific cellular uptake of folate-functionalized mFLAME

**Fig. 2:**
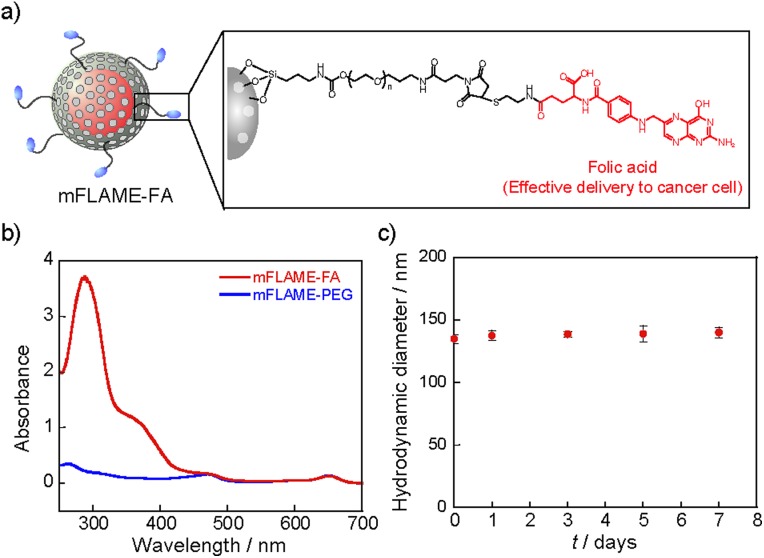
Physical properties of mFLAME–FA. (a) Chemical structure of mFLAME–FA. (b) UV-visible spectra of mFLAME–FA and mFLAME–PEG. (c) DLS analysis of mFLAME–FA followed over time at a storage temperature of 4 °C.

To demonstrate the efficient delivery of mFLAMEs for cancer therapy, we focused on the folate receptor, which is a 38 kDa glycophosphatidylinositol-linked membrane protein found on the surface of most solid tumors.^15^ Recently, it was reported that folate receptor-mediated uptake could be exploited to facilitate the entry of nanomaterials into cells.^16^ Hence, a folate-functionalized mFLAME nanoparticle (mFLAME–FA) was synthesized (Fig. 2a and Scheme S3†).

The *ζ*-potential value of mFLAME–FA was –45.1 mV, while that of the amino-functionalized mFLAME (mFLAME–NH_2_) was +18.4 mV. A PEGylated mFLAME (mFLAME–PEG) was also prepared as a control (Scheme S2†). The absorption spectra of mFLAME–PEG and mFLAME–FA revealed that only mFLAME–FA had absorption peaks at 280 and 362 nm, which were derived from the folate moiety (Fig. 2b). The mean diameters of mFLAME–FA in water 0, 1, 3, 5, and 7 days after synthesis were determined using DLS; almost no change in particle size was observed for 7 day (Fig. 2c). This result suggested that the mFLAME–FA possessed satisfactory stability in aqueous solutions. The cytotoxicity of the mFLAME nanoparticles towards KB cells was evaluated using the 3-(4,5-dimethylthiazol-2-yl)-2,5-diphenyltetrazolium bromide (MTT) assay; the cell viability was not affected by up to 0.32 mg mL^–1^ of PFCE (Fig. S3†), suggesting that the mFLAMEs were biocompatible and safe for *in vivo* applications.

**Fig. 3:**
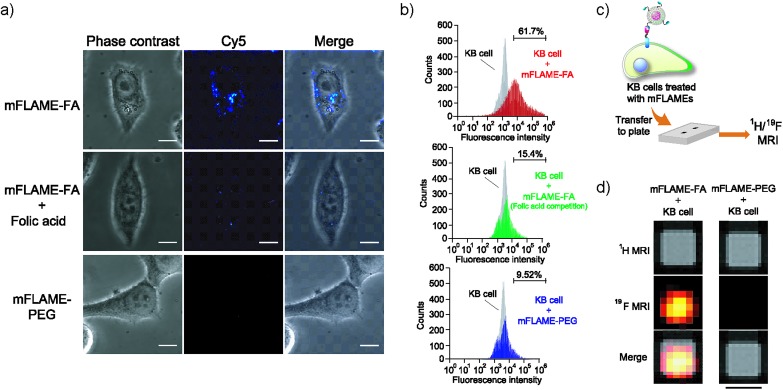
(a) Confocal laser scanning microscopy images of KB cells treated with mFLAME–FA or mFLAME–PEG with or without folic acid for 4 h. Measurement conditions: Cy5 was excited at 635 nm and detected at 660–760 nm. Scale bar: 10 μm. (b) Flow cytometric analysis of cellular uptake. The gray histograms show the distribution of non-treated cells. (c) Illustration of the experimental procedure for the MRI detection of mFLAME–FA in KB cells. (d) ^1^H/^19^F MR images of KB cells treated with mFLAME–FA or mFLAME–PEG. The matrix size was 64 × 64, slice thickness was 30 mm, and RARE factor was 16. *T*_E,eff_ and *T*_R_ were 40 ms and 1000 ms, respectively. The number of average was 128. The acquisition time is 34 min 20 s. Scale bar indicates 0.3 μm.

To further investigate the potential biomedical applications of mFLAMEs, their uptake into specific cells was evaluated *via* fluorescence imaging and ^19^F MRI. The expression of folate receptors on the cell surface was confirmed by a small-molecule imaging agent, fluorescein isothiocyanate–folic acid (FITC–FA) (Scheme S4†). As reported previously,^17^ folate receptors were overexpressed on KB cells and not expressed in A549 cells (Fig. S4†). The nanoparticle uptake into cells was visualized using confocal laser scanning microscopy (Fig. 3a). mFLAME–FA was internalized in KB cells. Furthermore, pre-incubation of free folic acid inhibited the specific uptake of mFLAME–FA. In contrast, the uptake of mFLAME–PEG was not observed with KB cells. In addition, almost no fluorescence was observed from A549 cells incubated with mFLAME–FA or mFLAME–PEG (Fig. S5†), indicating that folic acid on the mFLAME–FA surface was efficient for the internalization of the nanoparticle. The spot-like fluorescence image also indicated that mFLAME–FA was internalized *via* endocytosis, and nearly all nanoparticles remained in the endosomes (Fig. S6†).

Flow cytometry was also carried out in order to investigate the cellular uptake of mFLAME by KB cells (Fig. 3b). KB cells took up *ca.* 6.5-fold more mFLAME–FA than mFLAME–PEG. Upon treatment with free folic acid, the endocytosis of mFLAME–FA decreased by more than 75%.

The nanoparticle uptake into cells was also examined by ^19^F MRI. After mFLAME–FA was incubated with KB cells, the KB cells were transferred to a well of a microtiter plate and ^1^H/^19^F MRI experiments were performed (Fig. 3c). Fig. 3d shows MR images of the microtiter plate, including those of KB cells treated with mFLAME. ^19^F MRI signals were observed from mFLAME–FA, while no ^19^F MRI signal was observed from the control sample. Long acquisition time is required to acquire high contrast images. However, long acquisition time is not a disadvantage because the time scales of the dynamics of cellular uptake, DOX release, and apoptosis are not very fast. These results demonstrated that mFLAME could be used as a multimodal probe for ^19^F MRI and fluorescence imaging.

### Drug encapsulation and cellular toxicity of drug-loaded mFLAMEs

**Fig. 4:**
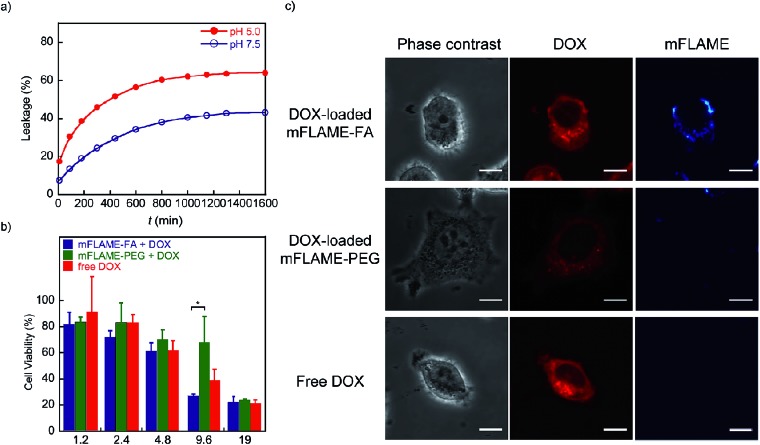
(a) Release profile of DOX-loaded mFLAME–FA in citric acid buffer (pH 7.5 and 5.0) at 37 °C [DOX] = 19 μg mL^–1^. (b) Concentration-dependent cell survival data of folate receptor positive-KB cells treated with free DOX or DOX-loaded mFLAME for 1 day. Values are expressed as the mean ± SE (error bars; *n* = 3, *P < 0.05). (c) Fluorescence imaging of KB cells treated with DOX-loaded mFLAME–FA, DOX-loaded mFLAME–PEG, and free DOX for 6 h [DOX] = 9.6 μg mL^–1^. Scale bar represents 10 μm.

To examine the potential of mFLAMEs for drug delivery, a chemotherapeutic agent and well-known anti-cancer drug,^18^ doxorubicin (DOX), was loaded into mFLAME. The loading amount of DOX in mFLAME was calculated using the difference in the UV spectra before and after loading. We then evaluated the release profiles of encapsulated DOX at different pH values by UV-visible absorption spectroscopy. Encapsulated-DOX was released gradually from mFLAME in a time-dependent manner (Fig. 4a). Interestingly, the release rates from mFLAME at pH 5.0 were faster than those at pH 7.5, which suggested that the surrounding pH affects the electrostatic interactions between mFLAME and DOX. The results indicated that mFLAME may have useful drug release abilities in the endosome and lysosome (pH 5.0–5.5), which have a lower pH than other cellular components, such as cytosol.^19^ mFLAME may also be useful for the treatment of cancer, because the pH of cancer cells is lower than that of normal tissues.

Next, DOX-loaded mFLAMEs were evaluated by assessing the viability of KB cells. Fig. 4b shows that DOX-loaded mFLAME–FA had a greater cytotoxic effect on KB cells as compared to that of mFLAME–PEG. In addition, the cytotoxicity of mFLAME–FA was greater than that of free DOX because mFLAME–FA was effectively internalized into KB cells owing to the folate ligand.

The cellular uptake of DOX-loaded mFLAME was analyzed by confocal laser scanning microscopy (Fig. 4c). The fluorescence of DOX was similar in KB cells incubated with DOX-loaded mFLAME–FA and in cells treated with free DOX. In contrast, a weaker fluorescence intensity was observed from cells incubated with DOX-loaded mFLAME–PEG, indicating that DOX-loaded mFLAME–FA exhibited strong cytotoxic effects on KB cells owing to efficient cellular internalization and drug release in the cells.

## Conclusions

We successfully developed a novel drug carrier based on a multimodal imaging agent, mFLAME, which consists of a PFCE core and mesoporous silica shell. The silica shell of mFLAME imparted various practical and useful properties such as dispersibility in water, chemical surface modifiability, biocompatibility, and efficient drug loading and release capacities. The PFCE core can serve as a highly sensitive ^19^F MRI contrast agent owing to the unrestricted mobility of multiple fluorine nuclei in the liquid-phase core. Since most MRI traceable-MSNs consist of doped magnetite nanoparticles or Gd^3+^ ion complexes for ^1^H MRI, it is often difficult to distinguish the distribution of the drug carrier due to the high background ^1^H MRI signals from water and lipids in living bodies. The ^19^F MRI detection system based on mFLAME has the potential to overcome this problem owing to the low background signals of ^19^F MRI.

Furthermore, because of the integrated features of the core–shell structure of ^19^F MRI and fluorescence imaging agents together with the drug delivery vehicle, mFLAME can be a useful tool for theranostic cancer treatment. By taking advantage of such properties, we demonstrated the dual modal detection of folate receptor-mediated cellular uptake *via* ^19^F MRI and fluorescence microscopy. More importantly, drug-doped mFLAME–FA exhibited adequate cellular uptake and drug release in folate receptor-overexpressing tumor cells. mFLAME should be tested in tumor-bearing mice, and simultaneous *in vivo* analysis of drug efficacy and biodistribution should be conducted in the near future.
