# Supplementary material for: Mesoporous silica nanoparticles for 19F magnetic resonance imaging, fluorescence imaging, and drug delivery
Source: Chem Sci. 2014 Dec 23;6(3):1986–90. doi: 10.1039/c4sc03549f (PMC5496002; doi:10.1039/c4sc03549f)
Supplement: Supplementary file 1 [file SC-006-C4SC03549F-s001.pdf]

Electronic Supplementary Material (ESI) for Chemical Science.
This journal is © The Royal Society of Chemistry 2014

Supporting Information

Mesoporous Silica Nanoparticles for ^{19}F Magnetic Resonance Imaging, Fluorescence Imaging, and Drug Delivery

Tatsuya Nakamura,[a] Fuminori Sugihara,[b] Hisashi Matsushita,[a] Yoshichika Yoshioka,[b] Shin Mizukami,[a,b] and Kazuya Kikuchi*[a,b]

[a]*Division of Advanced Science and Biotechnology, Graduate School of Engineering, Osaka University, 2-1 Yamadaoka, Suita, Osaka 565-0871, Japan*

[b]*Immunology Frontier Research Center, Osaka University, 3-1 Yamadaoka, Suita, Osaka 565-0871, Japan*

***Corresponding Author**

Correspondence should be addressed to K.K.
(kkikuchi@mls.eng.osaka-u.ac.jp)

1. Materials and Instruments

General chemicals were of the highest grade available and were supplied by Tokyo Chemical Industries, NOF Corporation, Wako Pure Chemical, or Aldrich Chemical Co. Sulfo-Cy5 NHS ester was purchased from Lumiprobe. 3-Aminopropyltriethoxysilane (APTES), *n*-cetylammonium bromide (CTAB), and tetraethyl orthosilicate (TEOS) were purchased from Tokyo Chemical Industry. Perfluoro-15-crown 5-ether (PFCE) was supplied by Wako Pure Chemical. MAL-$(CH_2)_2CONH(CH_2)_3$-O-PEG-COO-NHS (molecular weight [MW], 5,000; SUNBRIGHT® MA-050TS) and methoxy-PEG-$(CH_2)_5$-COO-NHS (MW 5,000, SUNBRIGHT® ME-050HS) were purchased from NOF Corp. Folic acid was purchased from Aldrich Chemical Co. The aforementioned reagents were used as received without further purification. KB cells were purchased from Health Science Research Resources Bank.

Nuclear magnetic resonance (NMR) spectra and longitudinal (T_1) and transverse (T_2) relaxation times were measured on a JEOL JNM-AL 400 instrument at 400 MHz for ^{1}H and at 100.4 MHz for ^{13}C NMR using tetramethylsilane as an internal standard, and at 376 MHz for ^{19}F NMR using sodium trifluoroacetate as an internal standard. Fluorescence spectra were recorded on a Hitachi F4500 spectrometer. The slit width was 5.0 nm for both excitation and emission. The photomultiplier voltage was 700 V. MRIs were recorded on a Bruker Avance II 500WB spectrometer equipped with a wide bore (89 mm), 11.7-T magnet, and a micro-2.5-imaging probe head operating at frequencies of 500 MHz for ^{1}H and 471 MHz for ^{19}F measurements. A volume coil with a 25 mm diameter was used. Image acquisition and processing were carried out using ParaVision software (Bruker BioSpin). TEM images were acquired using a HITACHI H-800 (200 kV). Particle size distribution and ζ-potential were measured via dynamic light scattering (DLS) on a particle analyzer (nano partica SZ-100, HORIBA). Fluorescence microscopic images were obtained using an Olympus FV10i-LIV confocal laser scanning microscope equipped with a ×60 lens. The emission filter sets used were Olympus BA490–540 for FITC-FA, and Olympus BA660–760 for mFLAME. All captured microscopic images were analyzed using ImageJ software. Flow cytometric analysis was performed using a cell analyzer EC 800 (Sony Biotechnology Inc.). The emission filter set used was BP700.

2. Synthesis and Experimental Procedures

Synthesis of mFLAMEs Sulfo-Cy5 NHS ester (2.0 mg) was reacted with APTES (10 μL) in ethanol (0.20 mL) in the dark for 1 d. CTAB (50 mg) was first dissolved in deionized water (6 mL). PFCE (30 μL) was added to the aqueous CTAB solution and the resulting solution was sonicated for 2 h at 50°C. The aqueous CTAB-PFCE solution was filtered through a 0.45-μm syringe filter to remove large aggregates or contaminants. The aqueous CTAB-PFCE solution was added to a solution of deionized water (23 mL) and 2 M sodium hydroxide (0.15 mL), and heated to 70°C with stirring.

Then, TEOS (0.25 mL), the Cy5-APTES solution (40 μL), and ethyl acetate (1.0 mL) were slowly added to the reaction solution and the resulting solution was stirred for 4 h. The synthesized materials were centrifuged (14,000 × *g*, 30 min) and washed with ethanol (20 mL) and H_2O (20 mL) three times and dispersed in 40 mL of isopropanol.

Synthesis of mFLAME-NH_2 mFLAMEs dispersed in isopropanol (40 mL) were heated to 80°C with stirring. Then, APTES (200 μL) was added slowly into the resulting solution and stirred for 4 h under Ar. The resulting materials were washed with ethanol three times. The CTAB surfactants were removed from the mesoporous silica nanoparticles by dispersing the mFLAME-NH_2 in a solution of ammonium nitrate (160 mg) and 95% ethanol (60 mL) and heating the mixture at 60°C for 1 h. The resulting materials were centrifuged (14,000 × *g*, 30 min) and washed with ethanol (20 mL) three times. mFLAME-NH_2 was subsequently dispersed in 20 mL of dimethylformamide (DMF).

Synthesis of mFLAME-COOH mFLAME-NH_2 dispersed in dry DMF (1.0 mL) was slowly added to a flask containing 2.0 M succinic anhydride in dry DMF under an Ar atmosphere. Anhydrous triethylamine (TEA) was added to the mixture, and the resulting mixture was stirred at 40°C for 24 h. The product was separated by centrifugation (14,000 × *g*, 4°C, 30 min) and washed three times with DMF (3.0 mL) and water (3.0 mL). Finally, mFLAME-COOH nanoparticles were dispersed in 3 mL of water.

Synthesis of mFLAME-Mal The mFLAME-NH_2 dispersed in dried DMF (10 mL) was heated to 40°C while stirring. Then, MAL-$(CH_2)_2$CONH$(CH_2)_3$-O-PEG-COO-NHS (0.40 g) and dried TEA (50 μL) were added and stirred for 2 d under an Ar atmosphere. The resulting materials were separated by centrifugation (14,000 × *g*, 30 min) and washed with DMF (5 mL) three times. mFLAME-Mal was dispersed in 10 mL of dimethyl sulfoxide (DMSO).

Synthesis of mFLAME-FA The synthesis of thiol-functionalized folic acid (FA-SH) was reported previously.[S1] mFLAME-Mal dispersed in DMSO (10 mL) was heated to 40°C under stirring. Then, FA-SH (50 mg) and dried TEA (50 μL) were added to the resulting solution and stirred for 2 d under an Ar atmosphere. The resulting materials were separated by centrifugation (14,000 × *g*, 30 min) and were washed with DMSO (5 mL) and H_2O (5 mL) three times. mFLAME-FA was dispersed in 5 mL of H_2O.

Synthesis of mFLAME-PEG mFLAME-NH_2 was dispersed in 10 mL of dried DMF and was heated to 40°C while stirring. Then, methoxy-PEG-$(CH_2)_5$-COO-NHS (0.40 g) and dried TEA (50 μL) were added to the resulting solution and stirred for 2 d under an Ar atmosphere. The resulting materials were separated by centrifugation (14,000 × *g*, 30 min) and were washed with DMF and H_2O three times. mFLAME-PEG was dispersed in 5 mL of H_2O.

Synthesis of FITC-NH_2 Ethylenediamine (600 μL, 9.0 mmol) was dissolved in 3.0 mL of DMF. Then, FITC (150 mg, 0.39 mmol) dissolved in DMF was added dropwise to the solution. The mixture was stirred for 12 h at room temperature (RT) and evaporated under reduced pressure. The

residue was washed three times with diethyl ether to produce FITC-NH_2 (yield (y.) 84%). ^{1}H NMR (400 MHz, DMSO-d_6) δ 2.75 (t, 2H), 2.88 (s, 1H), 3.05 (dd, 2H), 3.15 (s, 1H), 6.45–6.49 (m, 4H), 6.63–6.65 (m, 2H), 7.08 (d, 1H), 7.99 (s, 1H), 8.16 (s, 1H); MS (ESI$^+$) *m/z*: 450.07 (calculated for $[M+H]^+$): 450.11.

Synthesis of FITC-FA The synthesis of FA-NHS was reported previously.[S1] FITC-NH_2 (18 mg, 0.040 mmol) and FA-NHS (108 mg, 0.20 mmol) were dissolved in 6.0 mL of DMF. Triethylamine (29 μL, 0.20 mmol) was added to the solution. The mixture was stirred at RT for 12 h. After the reaction solution was evaporated, the product was purified by reverse-phase high-performance liquid chromatography and eluted with H_2O/acetonitrile containing 0.1% formic acid to yield FITC-FA (y. 18%). ^{1}H NMR (400 MHz, DMSO-d_6) δ 2.23 (t, 2H), 2.33 (s, 1H), 2.67 (s, 1H), 3.12 (m, 2H), 3.39 (s, 2H) 3.50 (s, 1H), 4.47 (d, 2H), 5.73 (s, 1H), 6.54–6.86 (m, 8H), 6.90–7.00 (m, 2H), 7.20 (d, 1H), 7.63 (d, 2H), 8.01–8.10 (m, 2H), 8.21 (s, 1H); HRMS (FAB$^-$) *m/z*: 871.2264 (calculated for $[M+H]^+$): 871.2324.

Fluorescence Imaging of Folate Receptors with FITC-FA KB cells and A549 cells were seeded in glass-bottom dishes and cultured under 5% CO_2 for 24 h. The cells were washed once with HBSS and treated with FITC-FA (5 μM) for 30 min. After a triple HBSS rinse, the fluorescence images were acquired by confocal laser scanning microscopy (CLSM).

Drug Release Studies DOX-loaded mFLAMEs were prepared in the same way as that used for cell cytotoxicity assessments. DOX-loaded mFLAMEs were dispersed in sodium citrate buffer (pH 7.5 and 5.0). At a certain time, an aliquot was separated by centrifugation (14,000 × *g*, 30 min) to remove the nanoparticles. The release of DOX from the pores was monitored via the absorbance of DOX at 480 nm. The total amount of DOX released was calculated from the absorbance.

***In Vivo* Experimental Procedure for MRI** Female mice (Balb/cA, 20 to 25 g bodyweight) were administered mFLAME-COOH (0.20 mL, C_{PFCE} = 2.6 mM) through tail-vein injection. For MRI measurements, mice were anesthetized using 2.0% isoflurane at a flow rate of 0.2 L/min and were subsequently scanned. All animal experiments were performed in compliance with the National Institutes of Health Guidelines for Care and Use of Laboratory Animals and were approves by the Osaka University Animal Care and Use Committee.

MRI Measurements The rapid acquisition with refocused echoes (RARE) method was used for ^{1}H and ^{19}F MRI. For ^{1}H MRI, the matrix size was 128 × 128, slice thickness was 2 mm, and RARE factor was 8. Repetition time (T_{R}) and effective echo time ($T_{\mathrm{E,eff}}$) were 1,000 ms and 31.4 ms, respectively. The number average was 1. For ^{19}F MRI, the matrix size was 64 × 64, slice thickness was 30 mm, and RARE factor was 16. T_{R} and $T_{\mathrm{E,eff}}$ were 1,000 ms and 80 ms, respectively. The number of average was 512.

^{19}F NMR Relaxation Time Measurements Nanoparticles were prepared in water containing 5% D_2O. The longitudinal relaxation time T_1 was measured by the inversion recovery method, and the

transverse relaxation time T_2 was measured by the spin-echo method.

DLS Measurements The particle size, size distribution, and ζ potential of the obtained nanoparticles were measured at 25°C with a 580 nm laser at a scattering angle of 90° for the DLS size measurements and 173° for the ζ potential measurements. For the DLS size measurements, mFLAME nanoparticles were suspended in water or ethanol. Suspensions of the materials in water were prepared for the ζ potential measurements.

Fluorescence Imaging of Cells Treated with mFLAMEs KB cells (folate receptor-positive cells) and A549 cells (folate receptor-negative cells) were seeded in glass-bottom dishes, and then cultured under 5% CO_2 for 24 h. For the free-folate competition studies, 1 mM folic acid was added to the medium and then incubated under 5% CO_2 for 1 h. The cells were washed once with Hank's balanced salt solution (HBSS) and incubated with mFLAMEs or doxorubicin (DOX)-loaded mFLAMEs (C_{PFCE} = 0.26 mM) for 4 or 6 h, respectively. The cells were treated with Lysotracker® blue (10 μM, Invitrogen) for 1 h after the treatment of the nanoparticles (Figure S6). After a triple HBSS rinse, the fluorescence images were acquired using CLSM.

Cellular Uptake Evaluated by Flow Cytometery KB cells were seeded into a 24-well plate and cultured under 5% CO_2 for 24 h. For the folate competition studies, 1 mM folic acid was added to the incubation medium and then incubated under 5% CO_2 for 1 h. The cells were washed once with HBSS and incubated with mFLAMEs for 4 h. After a triple HBSS rinse, the cells were harvested with trypsin-ethylenediaminetetraacetic acid (EDTA) and washed with HBSS. The cells were then re-suspended in phosphate-buffered saline (PBS) and analyzed using a flow cytometer.

^{19}F MRI Detection of Cellular Uptake KB cells were seeded in a 24-well plate and then cultured under 5% CO_2 for 24 h. The cells were washed once with PBS and incubated with mFLAMEs (C_{PFCE} = 0.52 mM) for 4 h. After a triple HBSS rinse, the cells were harvested with trypsin-EDTA and washed with HBSS. The cells (1.0 × 10^5 cells) were re-suspended in PBS and transferred to a customized 384-well plate for MRI measurements, and then ^{1}H/^{19}F MRI was performed.

Cell Cytotoxicity Evaluation of mFLAMEs 3-(4,5-Dimethylthiazol-2-yl)-2,5-diphenyltetrazolium bromide (MTT) assays were performed to evaluate the cytotoxicity of mFLAMEs. KB cells (5000 5,000 cells/well) were cultured in a 96-well plate under 5% CO_2 for 24 h. mFLAMEs (1.5 mg) were suspended in DOX solution and stirred for 24 h. The DOX-loaded mFLAMEs were separated by centrifugation (14,000 × *g*, 30 min) and were washed with H_2O. DOX-loaded mFLAMEs dispersed in the culture media with different concentrations of DOX (1.2, 2.4, 4.8, 9.6, and 19 μg/mL) were added to the wells. The cell culture medium is RPMI 1640 without phenol red and folic acid. The pH value was maintained at 7.2–7.4 via incubation in a 5% CO_2 atmosphere. After 24 h or 48 h incubation at 37 °C under 5% CO_2, the culture media was removed and MTT solution (100 μL) was added. After incubation for 4 h, the culture media was removed. Then, DMSO (100 μL/well) was added to each well and the absorbance at 590 nm was measured using a plate reader. The cell

cytotoxicity was finally expressed as the percentage of cell viability relative to that of the untreated control cells.

3. Supplementary Table and Figures

Materials	Hydrodynamic diameter/nm	ζ Potential/mV
PFCE emulsion	78 ± 5	51.0 ± 1.0
mFLAME	165 ± 8	-21.1 ± 2.5
mFLAME-NH_2	284 ± 23	18.4 ± 5.6
mFLAME-COOH	121 ± 5	-55.1 ± 2.3
mFLAME-Mal	134 ± 3	-40.1 ± 3.1
mFLAME-FA	142 ± 3	-45.1 ± 2.3
mFLAME-PEG	125 ± 6	-20.5 ± 2.1

Table S1. ζ Potential values and hydrodynamic diameters of PFCE emulsion and mFLAMEs.

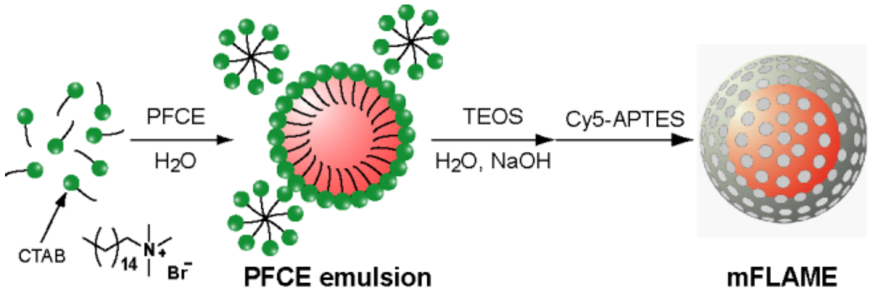

Scheme S1 Synthetic scheme of mFLAME.

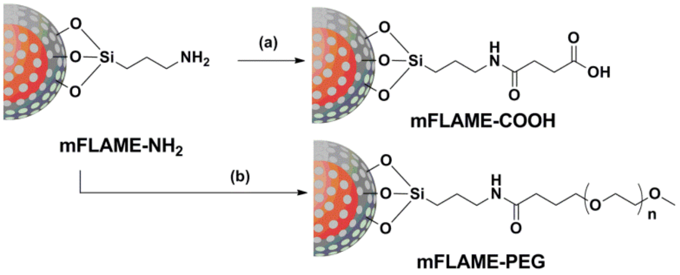

Scheme S2. Synthetic scheme of mFLAME-COOH and mFLAME-PEG. a) Succinic anhydride,

DMF, TEA; b) methoxy-PEG-$(CH_2)_5$-COO-NHS (MW 5,000), TEA, DMF.

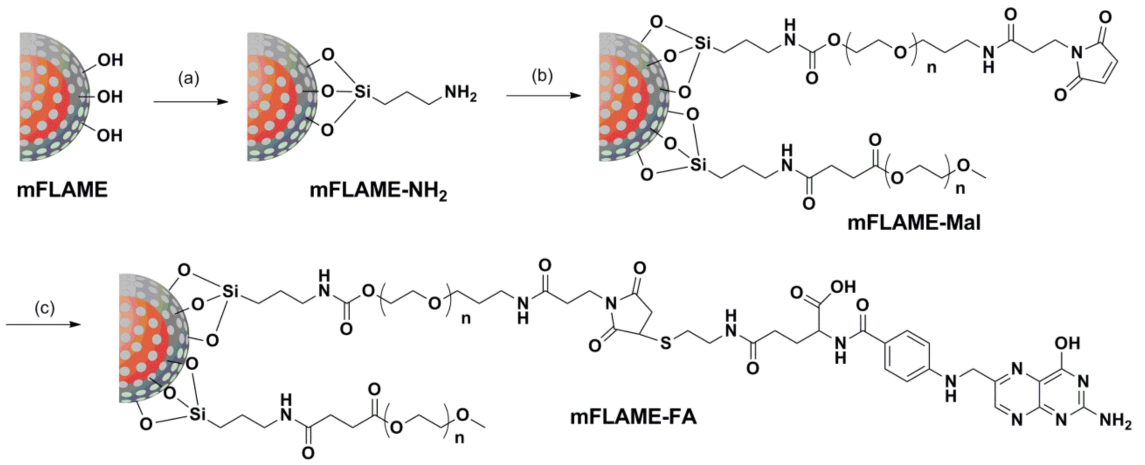

Scheme S3. Synthetic scheme of mFLAME-FA. a) APTES, 2-propanol; b) MAL-$(CH_2)_2$CONH$(CH_2)_3$-O-PEG-COO-NHS (MW 5,000), methoxy-PEG-$(CH_2)_5$-COO-NHS (MW 5,000), TEA, DMF; c) Folate-SH, TEA, DMSO.

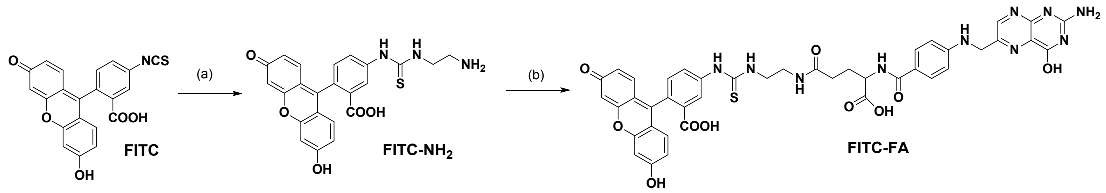

Scheme S4. Synthetic scheme of FITC-FA. a) ethylenediamine, DMF; b) FA-NHS, triethylamine, DMF.

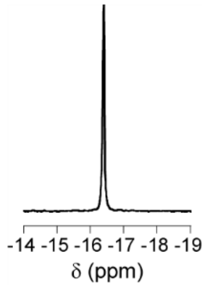

Figure S1. ^{19}F NMR spectra of mFLAME.

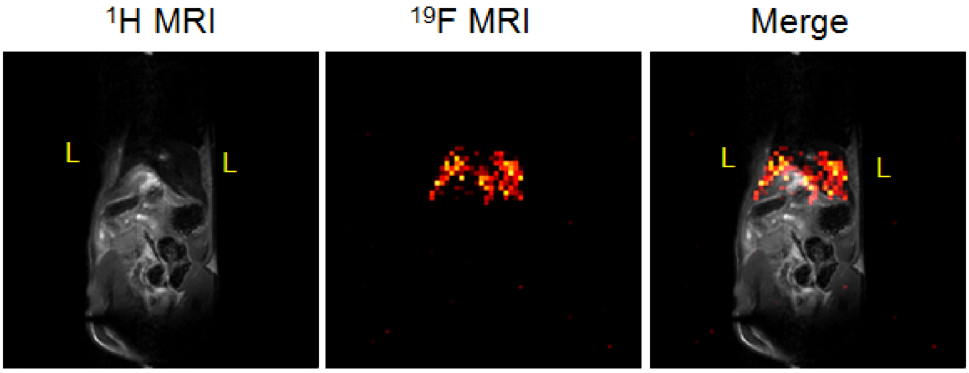

Figure S2. *In vivo* $^{1}H/^{19}F$ MRI of mFLAME-COOH in mouse. The position of the liver is represented as L. The matrix size was 64 × 64, slice thickness was 30 mm. $T_{E,eff}$ and T_R were 50.8 ms and 1000 ms, respectively. The number of average was 32. The acquisition time is 4 min 16 s.

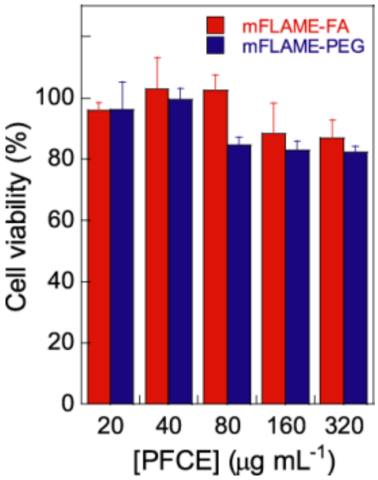

Figure S3. Cytotoxicity of mFLAME-FA and mFLAME-PEG towards cultured KB cells.

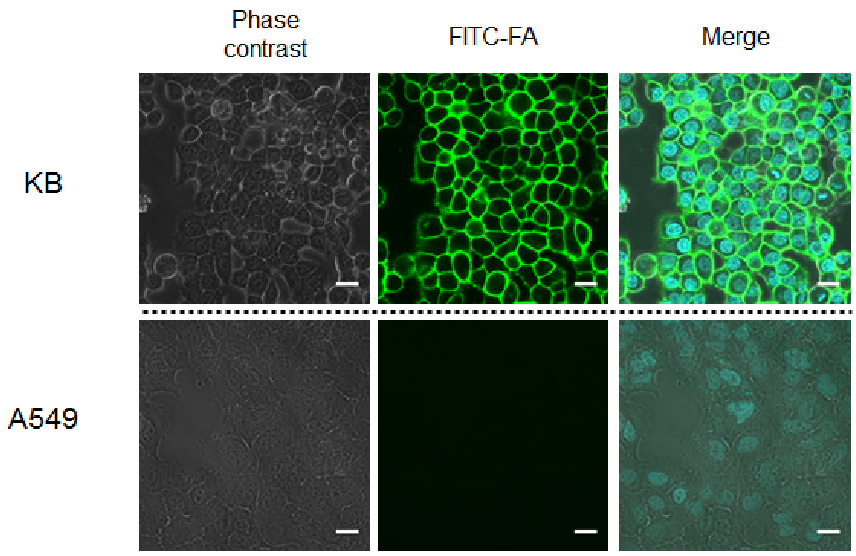

Figure S4. Fluorescence imaging of KB cells and A549 cells treated with FITC-FA. Scale bar represents 20 μm.

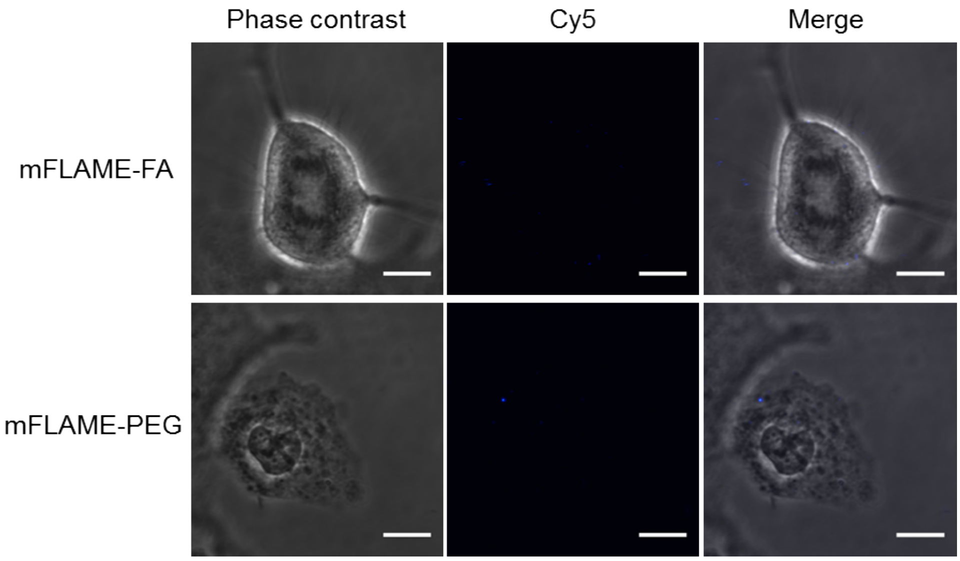

Figure S5. Fluorescence imaging of A549 cells treated with mFLAME nanoparticles. Scale bar represents 10 μm.

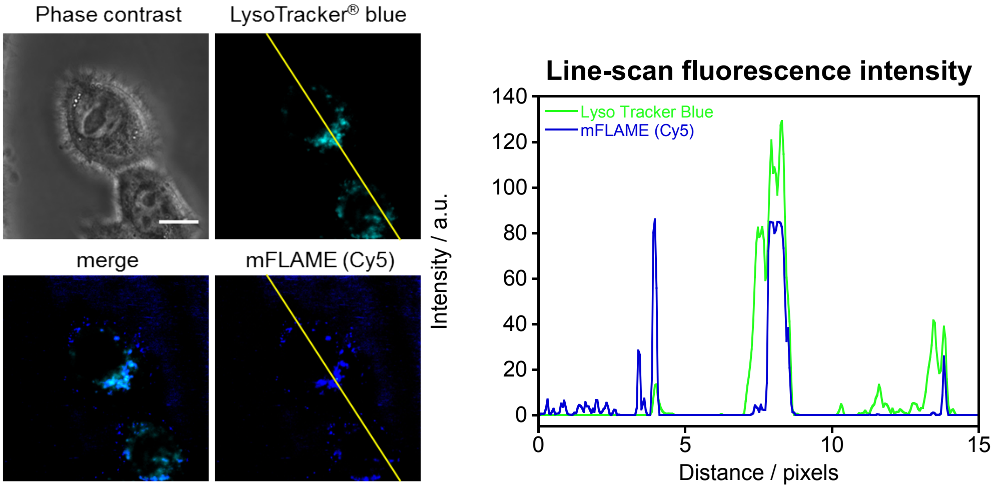

Figure S6. Fluorescence imaging of KB cells treated with mFLAME-FA and LysoTracker® blue. Scale bar represents 10 μm.

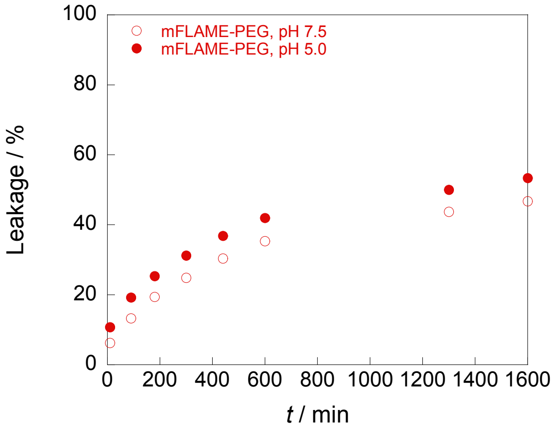

Figure S7. Release profile of DOX-loaded mFLAME-PEG in sodium citrate buffer (pH 7.5 and 5.0) at 37°C.

4. Reference

(S1) C. Zhang, S. Gao, W. Jiang, S. Lin, F. Du, Z. Li and W. Huang. *Biomaterials* 2010*,* **31**, 6075-6086.
